# 993 Validating a Novel Machine Learning Tool for Objective Measurement of Clinical Burn Scar Assessment

**DOI:** 10.1093/jbcr/iraf019.524

**Published:** 2025-04-01

**Authors:** Jordan Wong, Alexander Perry, Nidhi Gupta, Rakesh Joshi, Hannah Chan, Collin Hong, Joshua Wong

**Affiliations:** Alberta Health Services; University of Alberta; University of Alberta; Skinopathy; Skinopathy; Skinopathy; University of Alberta

## Abstract

**Introduction:**

Hypertrophic scars are a common and challenging sequelae of burn injuries, often carrying functional and psychosocial impairment. Unfortunately, there lacks standardization in clinical burn scar assessment. Typical scoring systems such as the Patient and Observer Scar Assessment Scale (POSAS) are based on subjective observations which leaves room for bias and interrater variability. Alternative methods that measure objective features (e.g., size, thickness, colour, and elasticity) show promise, though they require tools that are costly and time prohibitive. Herein we evaluate the validity of a novel objective measurement tool that utilizes smartphone photography and machine learning (ML) to generate quantitative scar measures.

**Methods:**

Burn patients recruited at a single centre underwent scar assessment using validated objective tools: ultrasound, pigmentation, and skin elasticity measurement devices. In tandem with the POSAS, we validated and trained our novel artificial intelligence-based tool with 2D colour images and video capture, with objective assessments as the best available gold standard.

**Results:**

Ongoing collection and analyses on 60 burn scars are being conducted. Preliminary machine learning size and pigmentation analyses are at par with the device assessments. POSAS scores positively correlate with objective and ML model measures. Promising preliminary data demonstrates that the ML model can perform size and pigmentation analysis. Additional training and alterations to the algorithm are required to produce measurements of lesion thickness and elasticity.

**Conclusions:**

This proof-of-concept study highlights the potential for ML models in burn scar assessment. Further development of this ML pipeline will allow for quick and accessible assessments of hypertrophic scars with mobile devices. Our approach could represent an alternative method for clinicians to track scar progression in patients with geographical barriers.

**Applicability of Research to Practice:**

With the apparent lack of accessible objective assessments for burn scars, this project addressed the barrier to provide clinicians with concrete measures. Accurately tracking the progression of burn scars in response to therapies can dictate patients care plan. As there is a widespread use of smart device photography in the charting of burn scars, incorporating ML technology proves feasible to the workflow. Once fully developed, loading our algorithm within an app or tool on smart devices will make objective burn scar assessments available and consistent across different sites.

**Funding for the Study:**

N/A

